# BTLA dysregulation correlates with poor outcome and diminished T cell-mediated antitumor responses in chronic lymphocytic leukemia

**DOI:** 10.1007/s00262-023-03435-1

**Published:** 2023-04-11

**Authors:** Christian Sordo-Bahamonde, Seila Lorenzo-Herrero, Alejandra Martínez-Pérez, Ana P. Gonzalez-Rodriguez, Ángel R. Payer, Esther González-García, Candelaria Aguilar-García, Sara González-Rodríguez, Alejandro López-Soto, Alejandra García-Torre, Segundo Gonzalez

**Affiliations:** 1 Department of Functional Biology, Immunology, Universidad de Oviedo, 33006 Oviedo, Spain; 2 Instituto Universitario de Oncología del Principado de Asturias (IUOPA), 33006 Oviedo, Spain; 3 Instituto de Investigación Sanitaria del Principado de Asturias (ISPA), 33011 Oviedo, Spain; 4 Department of Hematology, Hospital Universitario Central de Asturias (HUCA), 33011 Oviedo, Spain; 5 Department of Hematology, Hospital de Cabueñes, 33203 Gijón, Spain; 6 Department of Medicine, Universidad de Oviedo, 33006 PharmacologyOviedo, Spain; 7 Department of Biochemistry and Molecular Biology, Universidad of Oviedo, 33006 Oviedo, Spain; 8 Department of Immunology, Hospital Universitario Central de Asturias (HUCA), Oviedo, Spain

**Keywords:** CLL, Leukemia, T cell, BTLA, HVEM, Checkpoint

## Abstract

Patients with chronic lymphocytic leukemia (CLL) progressively develop marked immunosuppression, dampening innate and adaptive-driven antitumor responses. However, the underlying mechanisms promoting immune exhaustion are largely unknown. Herein, we provide new insights into the role of BTLA/HVEM axis promoting defects in T cell-mediated responses against leukemic cells. Increased expression of BTLA, an inhibitory immune checkpoint, was detected on the surface of CD4 + and CD8 + T lymphocytes in patients with CLL. Moreover, high levels of BTLA on CD4 + T cells correlated with diminished time to treatment. Signaling through BTLA activation led to decreased IL-2 and IFN-γ production ex vivo, whereas BTLA/HVEM binding disruption enhanced IFN-γ + CD8 + T lymphocytes. Accordingly, BTLA blockade in combination with bispecific anti-CD3/anti-CD19 antibody promoted CD8 + T cell-mediated anti-leukemic responses. Finally, treatment with an anti-BLTA blocking monoclonal antibody alone or in combination with ibrutinib-induced leukemic cell depletion in vitro. Altogether, our data reveal that BTLA dysregulation has a prognostic role and is limiting T cell-driven antitumor responses, thus providing new insights about immune exhaustion in patients with CLL.

**Supplementary Information:**

The online version contains supplementary material available at 10.1007/s00262-023-03435-1.

## Introduction

Within the last decade, the therapeutic landscape of patients with chronic lymphocytic leukemia (CLL) has been revolutionized by using small molecule inhibitors, including ibrutinib/acalabrutinib, idelalisib, and venetoclax [1, 2]. However, despite these recent advances in therapeutic protocols, CLL treatment is still challenging, and this hematological malignancy remains incurable. Treatment-associated toxicities and mutations affecting key genes have limited the clinical success of these approaches, highlighting the need for a deeper understanding of CLL pathogenesis that might improve patient management [3, 4].

CLL progression is associated with substantial NK cell and T cell exhaustion [5]. We and others have reported that increased expression of inhibitory immune checkpoints, such as LAG-3, ILT2, NKG2A, or TIGIT, plays an essential role in hampering the antitumor immune response [6–9]. Nonetheless, ICB-based therapeutical interventions targeting PD-1 failed to achieve clinical benefits in clinical trials in CLL, bringing to light the importance of unveiling the immunobiology of this malignancy [10].

B- And T-Lymphocyte Attenuator (BTLA), an inhibitory immune checkpoint expressed on B, T, and NK cells, has gained attention within the last few years [11]. HVEM stands as the binding partner for BTLA, it is characterized by a broader expression, since it can also be detected in hematopoietic, epithelial, and endothelial cells, and neurons [12]. HVEM works as a bidirectional switch, acting as a ligand for distinct co-stimulatory and co-inhibitory molecules, but also being able to activate its own signal transduction. This intricate network is crucial for the maintenance of homeostasis of the immune response [13]. Upon binding, HVEM provides pro-survival and proliferative signals through activation of NF-κB and AKT transcriptional pathways, whereas BTLA downregulates T cell-mediated responses [11, 14–17]. It has been previously described that BTLA engagement to HVEM results in defective T cell function. More specifically, tumor antigen-specific CD8 + T cells displayed enhanced cytokine production and proliferation upon BTLA blockade in melanoma models in vitro [18]. BTLA/HVEM axis dysregulation has been linked to poor outcome and diminished antitumor immune responses in a wide variety of cancers, including solid tumors (e.g. pancreatic adenocarcinoma, non-small-cell lung cancer) and those from the hematological origin (e.g. follicular lymphoma, CLL) [19–23]. Icatolimab, a first-in-class anti-BTLA monoclonal blocking antibody (mAb), has shown promising preliminary results in recent clinical trials in patients with advanced solid tumors [24]. Likewise, ongoing clinical trials are also being conducted to test the efficacy of icatolimab in hematological malignancies, including recurrent/refractory lymphoma (NCT04477772), although no results have been published to date.

In line with this, we have recently reported that BTLA/HVEM axis is deeply dysregulated on leukemic cells and NK cells from patients with CLL [25]. Importantly, enhanced BTLA expression on NK cells correlated to a shorter time to treatment (TTT) and diminished antitumor responses. Ex vivo treatment with anti-BTLA blocking mAbs restored, at least in part, NK cell-mediated anti-leukemic activity by promoting cytokine production and cytotoxicity, as well as antibody-dependent cytotoxicity (ADCC) in combination with the anti-CD20 antibody rituximab. Herein, we evaluate BTLA expression and function on T cells from patients with CLL.

## Results

### BTLA expression is increased on T lymphocytes from patients with CLL

**Fig. 1:**
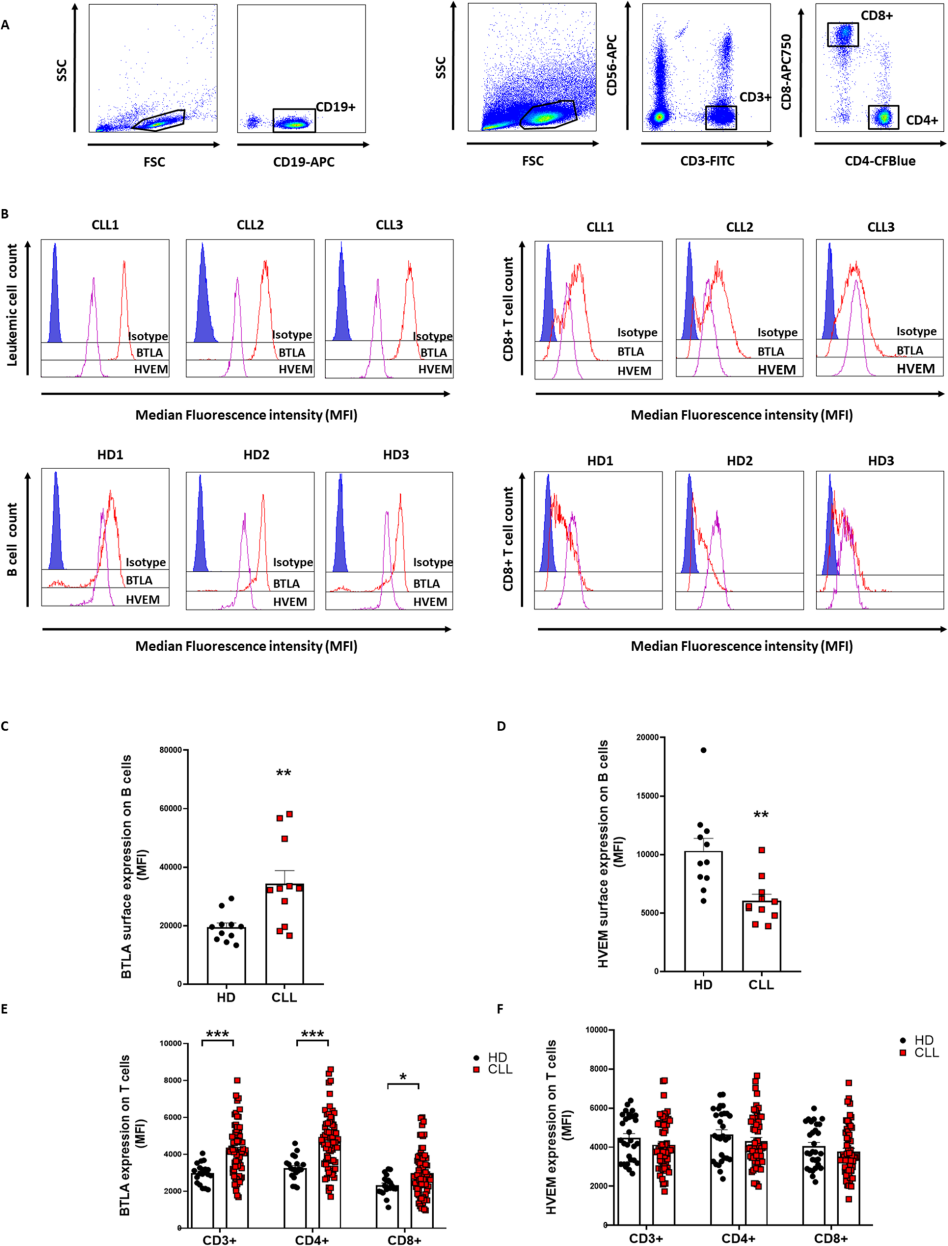
T cells from patients with CLL showed increased BTLA expression. BTLA and HVEM surface expression was evaluated on PBMCs from patients with CLL and HD by flow cytometry. **A** Gating strategy for leukemic cell and T cell subsets detection. **B** Representative histograms from three patients with CLL and three healthy donors (HD). **C**–**D** Comparison of BTLA and HVEM expression (MFI ± SEM) between leukemic cells and B cells from HD (n = 11). **E**–**F** Evaluation of surface BTLA/HVEM levels in T cell subsets from patients with CLL (n = 70) and HD (n = 20). *p < 0.05, **p < 0.01 and ***p < 0.001

We have previously reported that BTLA expression is increased on leukemic cells compared to their healthy counterpart [25]. These findings were confirmed in a new cohort of patients (n = 11) and HD (n = 11) upon phenotypic characterization of peripheral blood mononuclear cells (PBMCs) from patients with CLL and healthy donors (HD) (Fig. 1A–B, left panels). As expected, BTLA expression was increased in leukemic cells compared to B cells from HD (*p* = 0.0066), whereas HVEM levels were decreased (*p* = 0.0008) (Fig. 1C–D).

Surface BTLA/HVEM expression was next evaluated on T cells from 71 patients and 20 healthy donors by flow cytometry (Fig. 1A–B, right panels). BTLA expression was found to be significantly heightened in T cells from patients with CLL (Fig. 1E), however, no differences were detected regarding surface HVEM (Fig. 1F). Interestingly, BTLA levels were elevated in all the T cell subsets analyzed: total CD3 + (*p* < 0.0001), CD4 + (*p* < 0.0001), and CD8 + (*p* = 0.01) cells.

### BTLA expression on CD4 + T cells, but not on CD8 + T cells, correlates with a shorter time to treatment

**Fig. 2:**
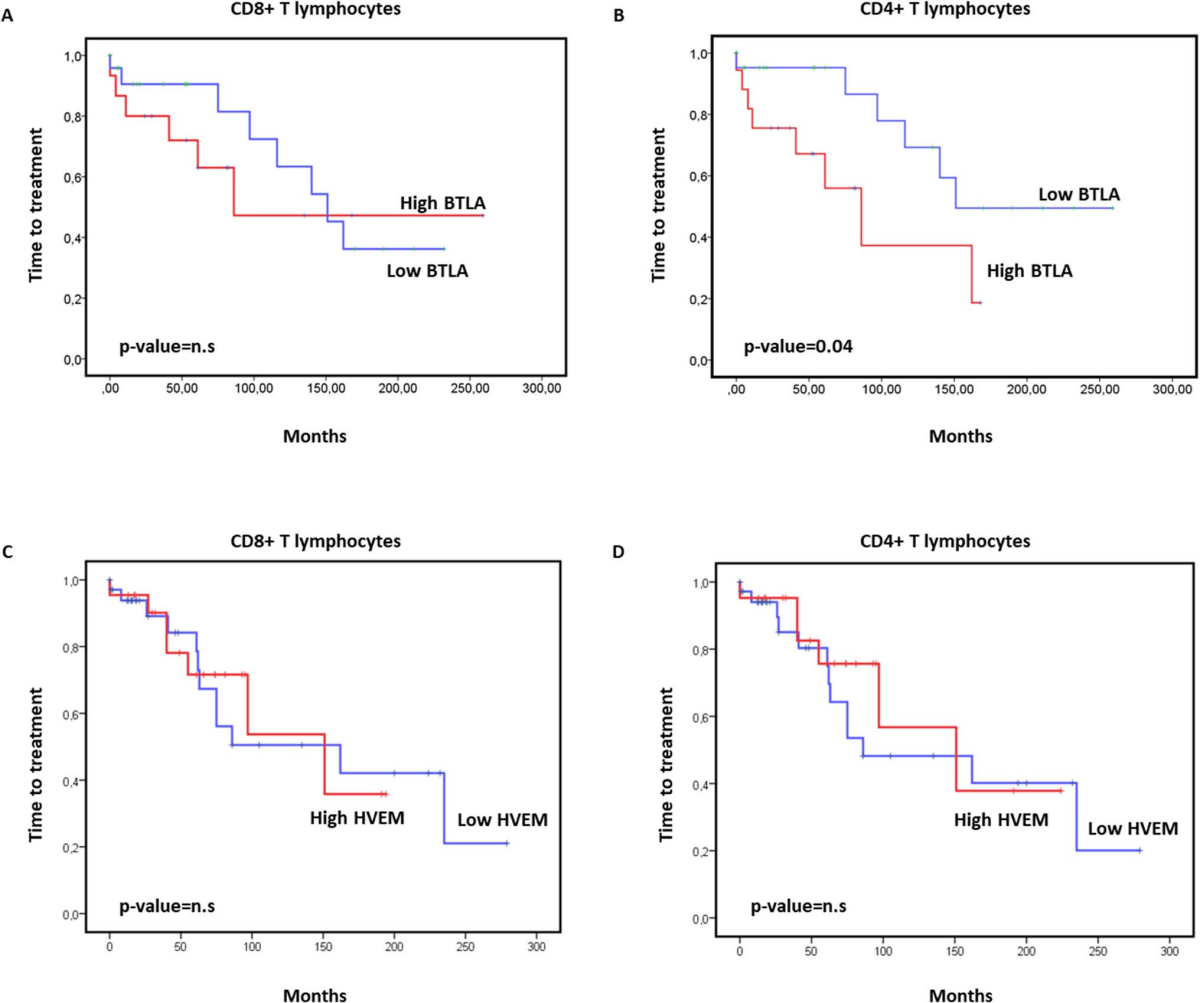
High BTLA expression on CD4 + T cells correlates with a shorter time to treatment in CLL. Kaplan–Meier survival analysis showing TTT in patients with CLL categorized by BTLA (**A**, **B**) or HVEM (**C**, **D**) levels on CD8 + T cells and CD4 + T cells. n.s. = not significant, *p* < 0.05

Next, we assessed whether the expression of BTLA/HVEM on T lymphocytes may predict a patient’s outcome. For this purpose, the impact on TTT of BTLA and HVEM surface expression on CD4 + and CD8 + T cells in our cohort was evaluated using Kaplan–Meier analysis. Our data revealed that high expression of BTLA on CD4 + T cells, but not on CD8 + T cells, correlated with diminished TTT (Fig. 2), suggesting that this inhibitory immune checkpoint might play a role in T cell exhaustion in this malignancy. Of note, no correlation between TTT and HVEM levels on CD4 + or CD8 + T cells was observed (Fig. 2C–D).

### BTLA blockade promotes cytokine production and T cell-mediated cytotoxicity

**Fig. 3:**
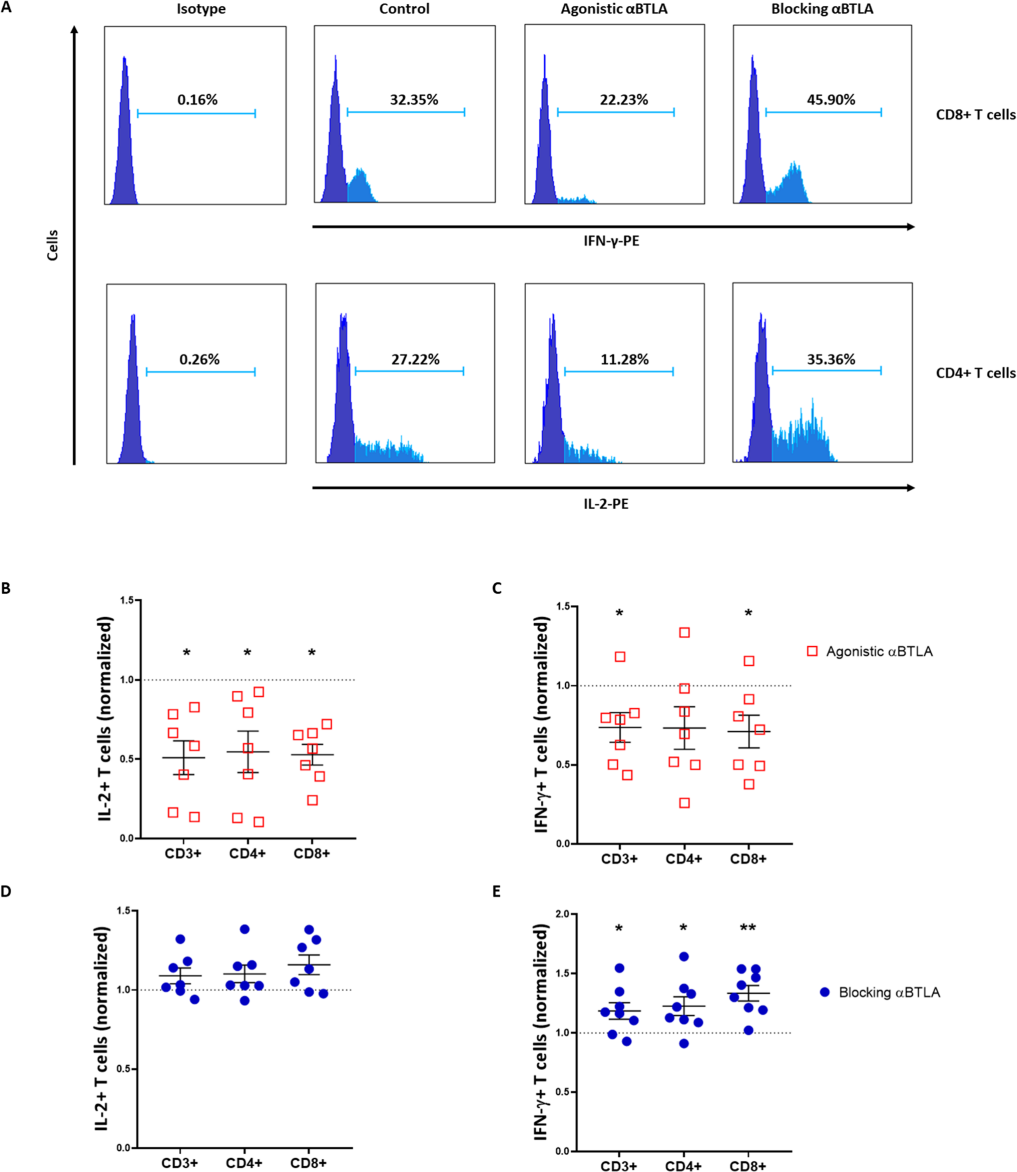
Ex vivo treatment with an anti-BTLA blocking antibody increases cytokine production by T cells in patients with CLL. **A** Representative histograms of IL-2 + CD4 + and IFN-γ + CD8 + T cells treated ex vivo with agonistic or antagonistic anti-BTLA mAbs. **B**–**E** Effect of BTLA signaling upon activation or blockade on IL-2 and IFN-γ production on the total T cell (CD3 + /CD56 −) and CD4 + /CD8 + T lymphocyte subsets (percentage normalized to control ± SEM). *p < 0.05, **p < 0.01

In order to elucidate whether BTLA dysregulation impinges on T lymphocyte-mediated responses, we first evaluated the relevance of BTLA on the immune production of cytokines with key roles in antitumor responses. More specifically, IFN-γ and IL-2 intracellular levels were assessed upon BTLA activation or blockade in PBMCs from 8 patients with CLL (Fig. 3A and Supplementary Figure S1). Treatment with agonistic anti-BTLA mAb led to a decreased percentage of IL-2 + CD3 + T cells (33.81 ± 5.57 vs. 21.71 ± 6.925, *p* = 0.01), IL-2 + CD4 + T cells (37.03 ± 5.518 vs. 23.87 ± 8.048, *p* = 0.01), as well as IL-2 + CD8 + T cells (18.05 ± 4.022 vs. 10.35 ± 3.051, *p* = 0.01) (Fig. 3B). In line with this, BTLA activation significantly reduced IFN-γ levels in these immune cell subsets (16.72 ± 2.758 vs. 11.46 ± 1.514, 8.13 ± 1.178 vs. 5.85 ± 1.99 and 41.91 ± 7.23 vs. 28.8 ± 4.17, respectively) (Fig. 3C). On the other hand, ex vivo treatment with antagonistic anti-BTLA mAb showed little effect on IL-2 production (Fig. 3D). However, IFN-γ intracellular levels were significantly augmented upon BTLA/HVEM axis disruption, mainly in cytotoxic CD8 + T cells (27.1 ± 6.01 vs. 37.32 ± 7.82, *p* = 0.007) (Fig. 3E).

### BTLA signaling disruption promotes T cell-mediated cytotoxicity

**Fig. 4:**
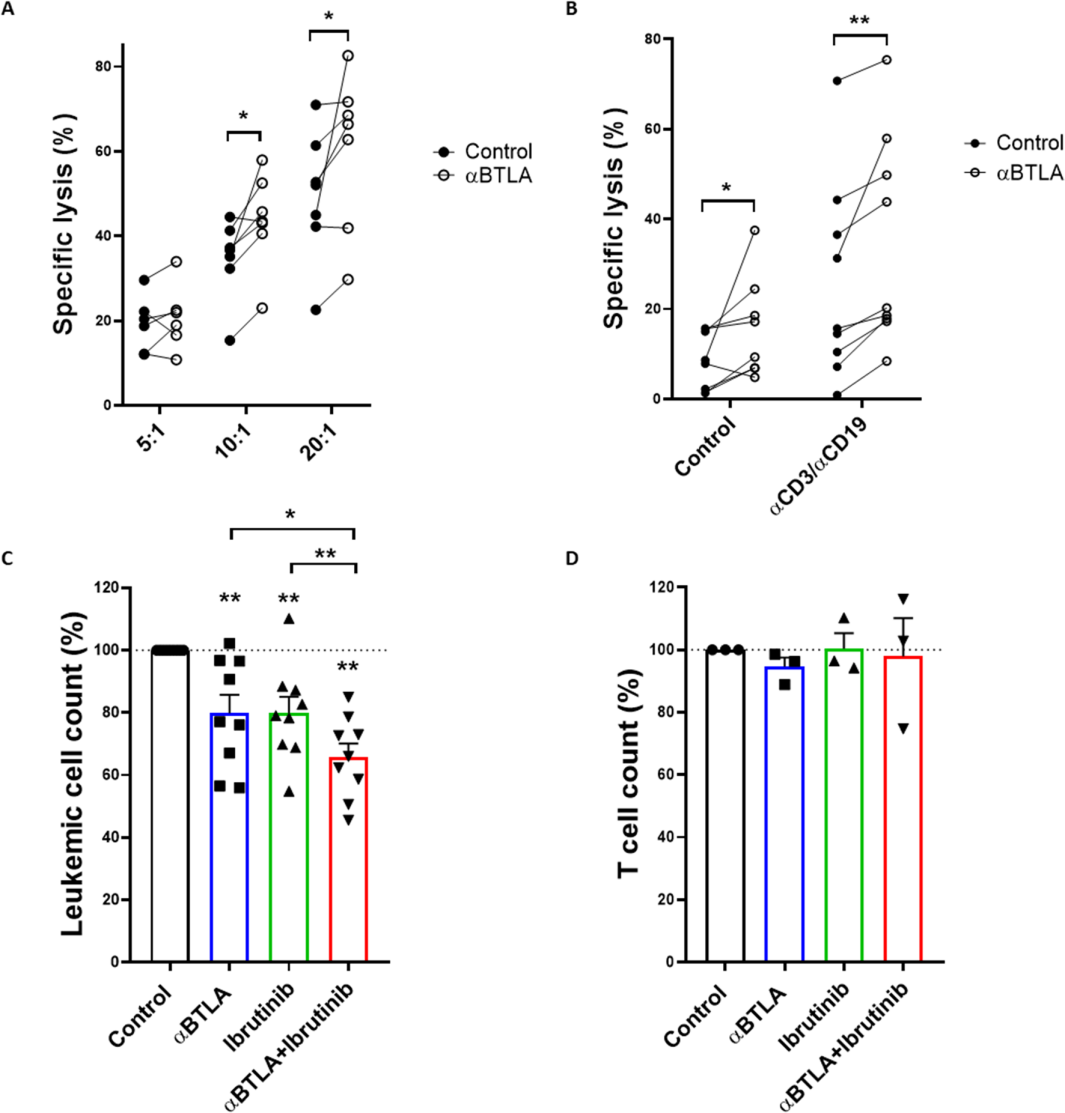
BTLA blockade promotes T cell-mediated anti-leukemic responses in CLL. **A** Cytotoxic activity was evaluated on stimulated HD-isolated CD8 + T lymphocytes treated with anti-BTLA mAb or isotype control and co-cultured with MEC-1 cell line as target cells at the indicated ratios (n = 7). Viability was measured by CD19/PI staining and flow cytometry. The effect of BTLA blockade on cellular cytotoxicity was evaluated in vitro by calcein-AM assay. **B** PBMCs from patients with CLL were treated with anti-BTLA mAb or isotype control and co-cultured with MEC-1 cell line at 50:1 (E:T) ratio. Where indicated, MEC-1 cells were pre-treated with 100 ng/mL bispecific anti-CD3/anti-CD19 antibody (n = 9). Absolute leukemic **C** and T cell **D** count was evaluated on PBMCs from patients with CLL (n = 9 and n = 3, respectively) upon treatment with 10 µg/mL anti-BTLA mAb or isotype control alone or in combination with 1 µM ibrutinib. *p < 0.05, **p < 0.01

Lastly, we evaluated whether treatment with anti-BTLA blocking mAbs specifically boosted T cell-mediated antileukemic cytotoxicity. For that purpose, stimulated HD-derived CD8 + T lymphocytes, pre-treated with blocking anti-BTLA mAb, were co-cultured with MEC-1 cell line at indicated E:T ratios (n = 7) (Fig. 4A). Noteworthy, MEC-1 cells display a similar phenotype to that of leukemic cells from patients with CLL, expressing high levels of surface BTLA (mean MFI: 61,629.7) and HVEM (mean MFI: 9515.4) (Supplementary Figure S1). The percentage of viable leukemic cells was analyzed by flow cytometry. As depicted in Fig. 4A, BTLA blockade significantly augmented the target cell lysis, supporting the role of BTLA/HVEM axis in dampening T cell-mediated cytotoxicity. Taking these results into account, we studied the impact of an anti-BTLA blocking mAb in combination with bispecific anti-CD3/anti-CD19 antibodies in CLL. PBMCs from 9 patients with CLL were treated with BTLA blocking antibodies for 72 h and, then, NK and T cell anti-leukemic responses against CLL-derived MEC-1 cells were evaluated (Fig. 4B). As we previously reported, BTLA blockade enhanced NK cell cytotoxicity [25]. In line with this, pre-treatment of MEC-1 target cells with bispecific anti-CD3/anti-CD19 antibodies increased specific leukemia cell lysis. Further, such cytotoxic activity was significantly heightened upon BTLA blockade.

Finally, we analyzed whether BTLA blockade might effectively work with BTK inhibitors routinely employed in the context of CLL. PBMCs obtained from 9 consecutive patients with CLL were treated ex vivo with anti-BTLA blocking mAb in combination with ibrutinib (Fig. 4C). BTLA blockade significantly reduced leukemic cell numbers after 72 h. Interestingly, a combination of both treatments significantly enhanced tumor cell depletion. Remarkably, no effect on T cell count was observed upon treatment with anti-BTLA mAb or ibrutinib (Fig. 4D).

## Discussion

The progression of CLL is tightly associated with growing immunosuppression affecting all compartments of the immune system promoting the development of secondary neoplasias and an increased risk of infections. Inhibitory immune checkpoint dysregulation has previously been associated with antitumor immune defects in CLL, including an altered cytokine profile and dampened NK cell and T cell cytotoxicity, which lead to lessened anti-leukemic responses [26]. In this line, we first reported the immunosuppressive and prognostic role of BTLA in CLL to gain insight into the potential of BTLA as a target for immunotherapy [25]. In this work, we further demonstrate that BTLA, but not HVEM, surface expression is increased on circulating CD4 + and CD8 + T cells from patients with CLL. Similar results have been detected on T cell subsets in other types of cancer, such as BTLA upregulation on peripheral CD4 + T cells in hepatocellular carcinoma or tumor antigen-specific CD8 + T cells from melanoma [15, 18, 27]. In line with our previous work, which disclosed that high BTLA surface expression on NK cells correlates with poor outcome, we herein report that increased levels of this immune checkpoint on CD4 + T cells are also associated with diminished TTT in patients with CLL. Notoriously, the prognostic value of BTLA expression has already been reported in other hematological malignancies. For instance, the presence of BTLA + T cells in the tumor microenvironment was associated with lower cytotoxic capability, advanced stage, and poor prognosis in diffuse large B-cell lymphoma [22].

Herein, immunosuppression and functional inhibition of T lymphocytes through BTLA was evaluated using agonistic and antagonistic anti-BTLA mAb. The activation of this inhibitory immune checkpoint decreased IL-2 and IFN-γ production, which is consistent with earlier studies [22, 28]. On the other hand, BTLA binding disruption with anti-BTLA blocking mAb partially restored cytokine production, enhancing the percentage of IFN-γ + T cells, but not IL-2 + T cells. Interestingly, dual BTLA/PD-1 blockade showed heightened IFN-γ levels and improved overall survival compared to monotherapies in murine models of glioblastoma [29]. Since BTLA has been reported to be co-expressed with several other immune checkpoints, whether the efficacy of BTLA blockade in monotherapy is related to inhibitory signaling through other receptors requires further investigations [22, 28]. In addition to cytokine production, BTLA tightly modulates cytotoxic responses driven by NK cells and CD8 + T lymphocytes as well, thus suggesting that this inhibitory checkpoint hinders innate and adaptive antitumor responses [22, 30, 31]. In CLL, and in consonance with our precedent work, the use of an anti-BTLA blocking mAb potentiated NK cell-mediated cytotoxicity. Moreover, the combination of BTLA blockade with bispecific anti-CD3/anti-CD19 antibodies boosted CD8 + T cell anti-leukemic activity, thus suggesting that BTLA upregulation is limiting antitumor responses.

Remarkably, and despite the disappointing results from initial clinical trials targeting PD-1/PD-L1, recent reports propose that a subset of patients might benefit from these therapies in combinatorial regimens. Treatment with pembrolizumab, alone or in combination BTK inhibitors, are currently ongoing in patients with high-risk CLL and those who underwent Richter transformation [10, 32]. Noteworthy, ibrutinib improves CLL-associated T cell dysfunction and downregulates BTLA expression on tumor cells without affecting its expression on T lymphocytes [33]. Here, we show that the combination of BTLA blockade with ibrutinib significantly increased leukemic cell depletion ex vivo without affecting T cell numbers. Altogether, these data suggest that BTLA/HVEM axis might favor immune exhaustion and tumor evasion in CLL.

BTLA and HVEM belong to an exceptionally complex network, since HVEM acts as a bidirectional switch, providing pro-survival signaling upon BTLA binding [17]. It is estimated that the *cis*-complex established between BTLA and HVEM represents approximately 80% of their surface reservoir on T cells, which prevents HVEM *trans* activation [34]. However, a recent study demonstrated that inhibitory signaling through BTLA plays a major role even in *cis* heterodimers, limiting T cell activation via HVEM [35]. Since BTLA expression is upregulated on CD4 + and CD8 + T lymphocytes from patients with CLL, whereas no changes on surface HVEM were detected on this immune subset, we hypothesize that BTLA may inhibit T cell-mediated responses through *cis* complexes as well as by *trans* interaction with HVEM on leukemic cells. The role of *cis* and *trans* BTLA/HVEM interplay favoring o limiting leukemic cell development has not been addressed in this work. Yet, HVEM/BTLA *trans* interaction among adjacent tumor cells in follicular lymphoma has been described to hinder tumor development [36]. Consequently, inactivating mutations or downregulation of these immune checkpoints might provide a mechanism for stimulating BCR-associated mitogenic signals in lymphoma cells [36]. Whether surface BTLA and HVEM expression may act as a tumor suppressor in leukemic cells in CLL deserves further investigation.

In conclusion, our study, despite its limitations, demonstrates that BTLA/HVEM axis is highly dysregulated on T cells from patients with CLL and increased BTLA expression on CD4 + T lymphocytes correlates with shorter TTT. BTLA blockade promotes anti-leukemic responses driven by NK cells and T cells by boosting cytokine production and cytotoxicity. Accordingly, herein, we provide the rationale for further investigating novel anti-BTLA mAbs such as icatolimab, and the clinical relevance of the BTLA/HVEM axis in CLL alone or in combination with BTK inhibitors.

## Materials and methods

### Samples

71 consecutive non-treated patients with CLL were included in the study. Peripheral blood samples from patients with CLL were obtained from Hospital Universitario Central de Asturias (HUCA) and Hospital Universitario de Cabueñes (Table 1), whereas samples from HD were provided by Centro Comunitario de Sangre y Tejidos de Asturias. Written informed consent following the Declaration of Helsinki was obtained from all individuals with approval from the local ethics committee (case-19042016). The median follow-up from the diagnosis of patients was 74 months. PBMCs were obtained by Ficoll density gradient centrifugation (Histopaque®-1077).

**Table 1:** Clinical characteristics of patients with CLL

	Patients (n = 71)	%
Age		
Years (mean)	66.7	
Sex		
Female	31	43.6
Male	40	56.4
Rai Stage		
0	37	52.1
I-II	21	29.5
III-IV	13	18.3
Binet Stage		
A	51	71.8
B	11	15.4
C	9	12.6
Cytogenetic abnormalities (FISH)		
No alterations	16	22.5
del(13q)	19	26.7
del(11q)	4	7.0
del(17p)	5	5.6
Trisomy 12	7	9.8
Others	19	26.7
*IGHV* status		
Mutated	43	60.5
Unmutated	15	21.1
Unknown	13	18.3
Progression		
Stable disease	45	63.3
Progressive disease	26	36.6

### Phenotypical analyses

BTLA and HVEM expression on B cells and T lymphocytes from patients with CLL and HD was assessed in fresh by flow cytometry using the antibodies listed below (Supplementary Table 2).

### Intracellular cytokine measurement

Intracellular cytokine staining was performed as previously described by our group [37]. Patient-derived PBMCs were cultured with anti-BTLA blocking antibody (clone 3B1, murine Ig G_1,_ kindly provided by Genentech) or proper isotype control (murine IgG_1_ kindly provided by Dr. Juan Ramón de los Toyos González, Universidad de Oviedo, Oviedo, Spain) for 72 h at 10 µg/mL. For BTLA activation experiments, PBMCs were cultured in 96-well plates coated with 10 µg/mL agonistic anti-BTLA antibody (clone MIH26, Biolegend, San Diego, CA, USA) or isotype control (clone MG2a-53, Biolegend, San Diego, CA, USA) for 24 h. PBMCs were stimulated with 50 nM PMA and 1 µg/mL ionomycin for 4 h and brefeldin A was added after 1 h of incubation (Biolegend, San Diego, CA, USA). Right afterward, immune subsets were stained as mentioned above and BD Cytofix/Cytoperm Fixation/Permeabilization Kit (BD Biosciences, BD Biosciences, San Jose, CA, USA) was employed according to the manufacturer’s protocol. IFN-γ and IL-2 production by T lymphocytes was evaluated using anti-IFN-γ-PE or anti-IFN-γ-PercP/C5.5 (clone 4S.B3, Biolegend, San Diego, CA, USA) and anti-IL-2-PE (clone MQ1-17H12, Biolegend, San Diego, CA, USA) and flow cytometry.

### Determination of NK and T cell-mediated cytotoxicity

NK and T cell cytotoxic activity was measured by the calcein-AM assay as previously described [38]. Briefly, PBMCs from patients with CLL were treated with 10 μg/mL anti-BTLA (clone 3B1, Genentech) or isotype control for 72 h. CLL-derived MEC-1 cell line (ATCC) was employed as target cells and stained with 10 µM calcein-AM (Biolegend). Then, tumor cells were co-cultured with PBMCs at a 50:1 effector: target (E:T) ratio for 4 h. Right afterward, calcein release was measured on a Varioskan™ LUX multimode microplate reader. In order to evaluate allogeneic T cell-mediated cytotoxicity, MEC-1 cell line was pre-incubated with 100 ng/mL bispecific anti-CD3/anti-CD19 (Invivogen) for 45 min.

For in vitro evaluation of BTLA blockade on T cell cellular cytotoxicity, HD CD8 + T lymphocytes were isolated using MojoSort™ Human CD8 T Cell Isolation Kit (Biolegend). Purified CD8 + T cells were stimulated with ImmunoCult™ beads (Stemcell) in the presence of blocking anti-BTLA mAb (clone 3B1, Genentech) or isotype control (10 μg/mL) for 7 days. Stimulated T cells were then incubated with MEC-1 cell line for an additional 72 h at indicated E:T ratios and the viability of tumor cells was evaluated by CD19-APC and PI staining (Immunostep). Basal apoptosis was measured by incubating target cells alone and specific lysis was calculated as previously detailed [39].

### Absolute leukemic cell count

PBMCs from patients with CLL were treated with anti-BTLA blocking antibody (clone 3B1, Genentech) or isotype control (10 µg/mL) alone or in combination with 1 µM ibrutinib (MedChemExpress) or vehicle (DMSO) for 72 h. Then, PBMCs were stained for leukemic/T cell identification, and an equal volume of cell count reference microbeads was added to each condition (Sigma-Aldrich). 5 × 10^3^ reference beads were acquired in each well by flow cytometry and absolute leukemic cell count was determined.

### Statistics

The normality was tested by the Shapiro–Wilk test. The relationship between continuous and categorical variables was evaluated by Mann–Whitney U-test. Wilcoxon Matched-Pairs Signed Ranks test was used for intra-group comparisons. For time-to-treatment analysis, considering time to treatment as the period from diagnosis to the first therapeutic intervention, Kaplan–Meier curves were plotted, and each group was compared by log-rank test using SPSS v23.0 software. Patients were cataloged using the median value as the cutoff level. Patients with a follow-up ≤ 1 year were excluded from the Kaplan–Meier analysis. *p*-values ≤ 0.05 were considered statistically significant.

## Supplementary Information

Below is the link to the electronic supplementary material.

Supplementary figure S1. **A** Representative dot plots of IL-2+ and IFN-γ+ CD4+ and CD8+ T cells treated ex vivo with agonistic or antagonistic anti-BTLA mAbs. **B** Representative histograms of BTLA and HVEM surface expression on MEC-1 cell line analyzed by flow cytometry (TIF 506 KB)

Supplementary file2 (DOCX 20 kb)
