# Supplementary figures and images for: BTLA dysregulation correlates with poor outcome and diminished T cell-mediated antitumor responses in chronic lymphocytic leukemia
Source: Cancer Immunol Immunother. 2023 Apr 11;72(7):2529–39. doi: 10.1007/s00262-023-03435-1 (PMC10264494; doi:10.1007/s00262-023-03435-1)

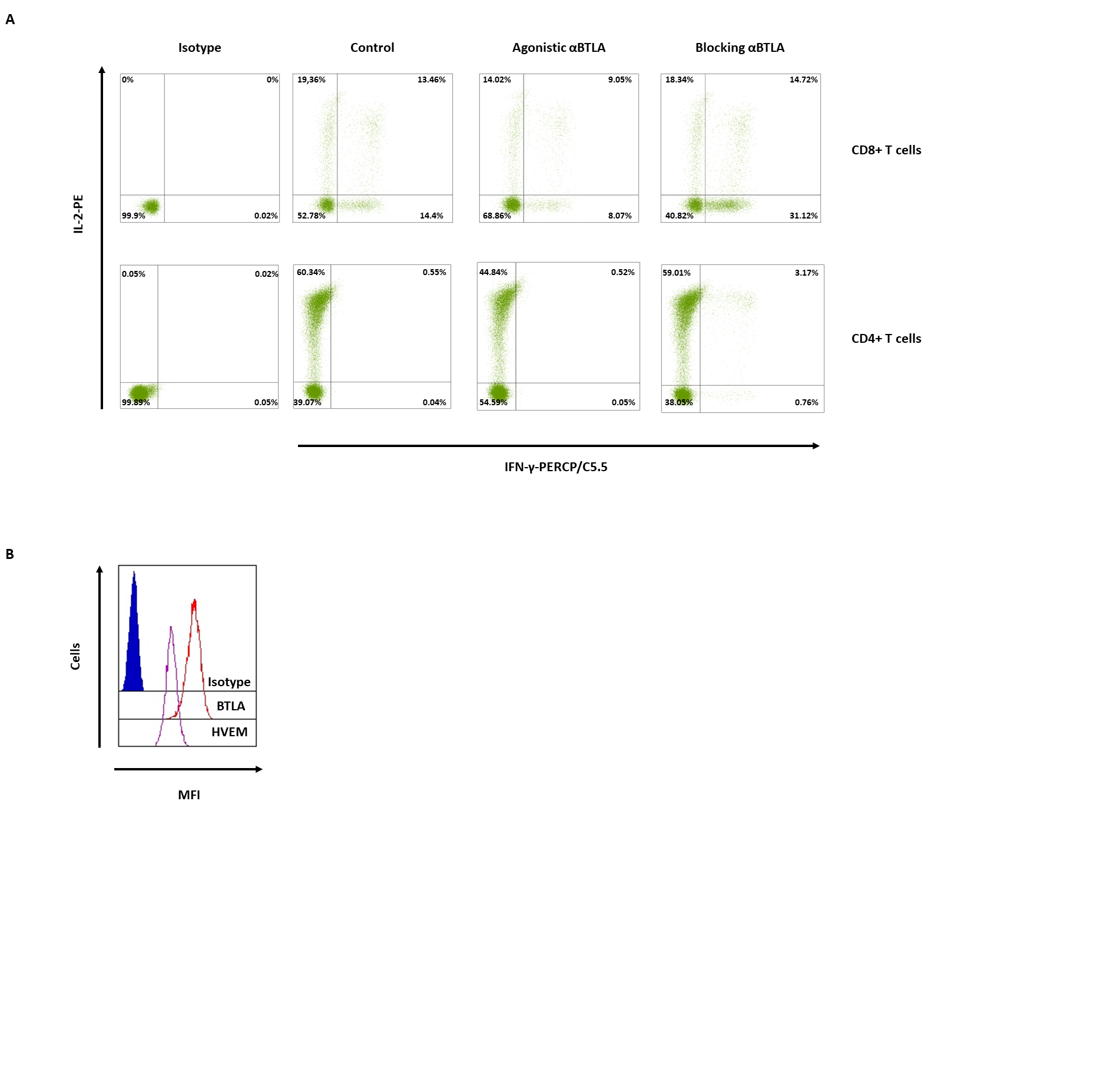

Supplement: Supplementary file 1 — Supplementary figure S1. A Representative dot plots of IL-2+ and IFN-γ+ CD4+ and CD8+ T cells treated ex vivo with agonistic or antagonistic anti-BTLA mAbs. B Representative histograms of BTLA and HVEM surface expression on MEC-1 cell line analyzed by flow cytometry (TIF 506 KB) [file 262_2023_3435_MOESM1_ESM.tif]
