# Supplementary material for: BTLA dysregulation correlates with poor outcome and diminished T cell-mediated antitumor responses in chronic lymphocytic leukemia
Source: Cancer Immunol Immunother. 2023 Apr 11;72(7):2529–39. doi: 10.1007/s00262-023-03435-1 (PMC10264494; doi:10.1007/s00262-023-03435-1)
Supplement: Supplementary file 2 — Supplementary file2 (DOCX 20 kb) [file 262_2023_3435_MOESM2_ESM.docx]

Table 2. List of antibodies

| Antibodies | Clone | Company |
| --- | --- | --- |
| CD19-APC | 33-2A3 | Immunostep |
| CD56-APC | QA17A16 | Biolegend |
| CD3-FITC | UCHT1 | Biolegend |
| CD4-CFBlue | HP2/6 | Immunostep |
| CD8-APC750 | 143-44 | Immunostep |
| BTLA-PE | MIH26 | Biolegend |
| HVEM-PE | 122 | Biolegend |
| IL-2-PE | MQ1-17H12 | Biolegend |
| IFNγ-PE | 4S.B3 | Biolegend |
| IFNγ-PercP/C5.5 | 4S.B3 | Biolegend |
